# Magnetic-field-induced dielectric behaviors and magneto-electrical coupling of multiferroic compounds containing cobalt ferrite/barium calcium titanate composite fibers

**DOI:** 10.1016/j.jallcom.2018.01.081

**Published:** 2018-04-05

**Authors:** Deqing Zhang, Junye Cheng, Jixing Chai, Jiji Deng, Ran Ren, Yang Su, Hao Wang, Chunqing Ma, Chun-Sing Lee, Wenjun Zhang, Guang-Ping Zheng, Maosheng Cao

**Affiliations:** a School of Materials Science and Engineering, Qiqihar University, Qiqihar 161006, China; b Guangdong Provincial Key Laboratory of Micro/Nano Optomechatronics Engineering, College of Mechatronics and Control Engineering, Shenzhen University, Shenzhen 518060, China; c Center of Super-Diamond and Advanced Films (COSDAF), City University of Hong Kong, 999077, Hong Kong; d Department of Mechanical Engineering, Hong Kong Polytechnic University, Hung Hom, Kowloon, Hong Kong; e School of Materials Science and Engineering, Beijing Institute of Technology, Beijing 100081, China

**Keywords:** Dielectric property, Cobalt ferrite, Barium calcium titanate, Aligned composite fibers, Electrospinning

## Abstract

Multiferroics have broad application prospects in various fields such as multi-layer ceramic capacitors and multifunctional devices owing to their high dielectric constants and coupled magnetic and ferroelectric properties at room temperature. In this study, cobalt ferrite (CFO)/barium calcium titanate (BCT) composite fibers are prepared from BCT and CFO sols by an electrospinning method, and are then oriented by magnetic fields and sintered at high temperatures. The effects of magnetic fields and CFO contents on the nanostructures and magnetoelectric properties of the composites are investigated. Strong coupling between magnetic and ferroelectric properties occurs in CFO/BCT composites with magnetic orientation. More interestingly, the dielectric constants of CFO/BCT composites with magnetic orientation are found to be enhanced (by ∼1.5–3.5 times) as compared with those of BCT and CFO/BCT without magnetic orientation. The boost of dielectric constants of magnetic-field orientated CFO/BCT is attributed to the magneto-electrical coupling between CFO and BCT, where the polar domains of BCT are pinned by the orientated CFO. Therefore, this work not only provides a novel and effective approach in enhancing the dielectric constants of ceramic ferroelectrics, which is of tremendous value for industrial applications, but also elucidates the interaction mechanisms between ferromagnetic phase and ferroelectric phase in multiferroic compounds.

## Highlights

• The BCT/CFO composite fibers are prepared by a facile synthesis route.
• BCT/CFO with magnetic orientation treatment shows strong magneto-electrical coupling.
• The dielectric constants of CFO/BCT are enhanced by magnetic orientation treatment.
• The mechanisms of magneto-electrical coupling between CFO and BCT are elucidated.

## Graphical abstract

**Figure:**
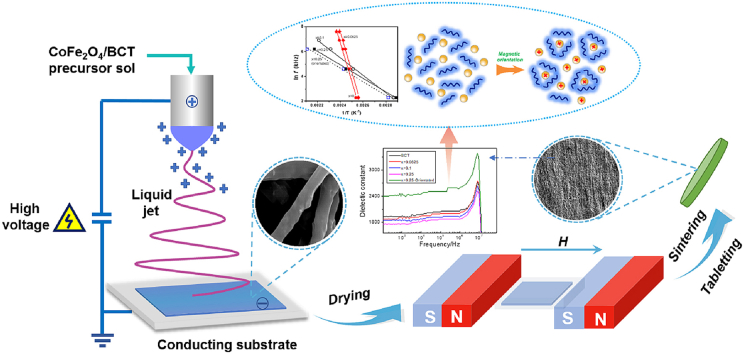

## 1 Introduction

With the rapid advancement of electronics, electronic materials are currently developed towards miniaturization, multifunction and integration to satisfy different application circumstances and requirements [1], [2]. Multiferroics, which refer to the functional materials that possess two or more properties of the ferromagnetism, ferroelectricity and ferroelasticity over a certain range of temperature, are important members of electronic materials family because of their promising applications in advanced electronic devices [3], [4], [5]. Among multiferroics, functional materials possessing electro-magneto-coupling are known as magnetoelectric (ME) materials [6], [7], which have wide and important applications in the fields of microwave devices and sensors for magnetic field detection [3], [4], [5], [8], [9], [10].

Recently, composite materials combining multi-phase ferroelectric and ferromagnetic materials for magnetoelectric applications are of great interest [11], [12], [13], [14], [15]. Particularly, it has been of great interest in achieving strong magnetoelectric coupling by combining ferroelectric barium titanate (BTO) with magnetic cobalt ferrite (CFO) [16], [17], [18]. BTO is one of the most typical ferroelectric materials with high dielectric constant and low dielectric loss [19]. As a widely used soft magnetic material, CFO is a spinel ferrite which exhibits excellent electromagnetic properties, high chemical stability, magneto-crystalline anisotropy and large magnetostriction coefficient [20]. Nevertheless, the desired magnetoelectric coupling properties of the composites have to be adjusted by volume ratio of the constituent phases, as well as the degree of interconnectivity and the properties of interface between BTO and CFO [16], [17], [18], [21].

The BTO/CFO multi-phase magnetoelectric materials have been prepared by various methods. Stenaciu et al. prepared BTO/CFO multi-phase magnetoelectric materials by the spark plasma sintering (SPS) method and investigated their magnetoresistance effect [22]. They found that the magnetic performance of the multi-phase material at 150 K reached an optimum at a CFO content of 30%. Raidongia and Kalyan prepared the core–shell BTO/CFO composite nanomaterial and studied the dielectric properties of core–shell nanoparticles under magnetic fields [23]. They concluded that the dielectric constant decreased with the application of magnetic fields. Zhang et al. prepared the BTO/CFO nanocomposite film with good ferroelectricity and ferromagnetic properties at room temperature via a method combining sol–gel and electrophoretic deposition [24]. However, the barium titanate crystals in the nanocomposites exhibited a tetragonal to orthorhombic phase transformation at ∼9 °C and might damage the composite, causing adverse effects in the applications of BTO/CFO [25]. Therefore, in this work, calcium is first doped into BTO to prepare barium calcium titanate (BCT) crystals, suppressing the phase transformation of BTO in the magnetoelectric composite.

Furthermore, fiber-like BCT/CFO magnetoelectric composites are prepared by electrospinning which is a facile route in preparing polymeric and inorganic nanofibers [26], [27], [28]. It is envisaged that the huge specific surface area of BCT/CFO nanofibers could enhance the surface or interfacial effects on the magnetoelectric coupling and thus the magnetoelectric properties of the BCT/CFO nanofibers could be improved [29], [30].

**Scheme 1:**
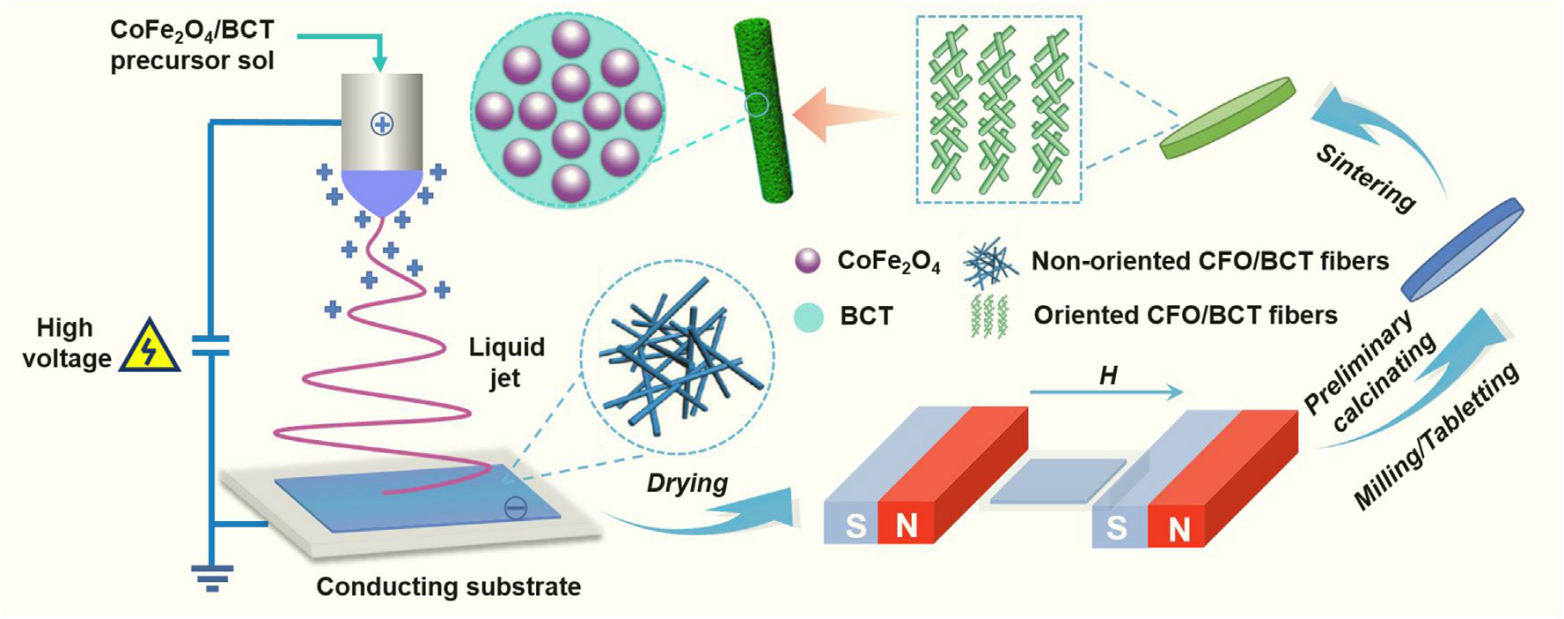
Schematic illustration of preparation of the aligned BCT/CFO nanocomposite fibers via a magnetic field induction approach.

The preparation route for the magnetoelectric nanofibers is illustrated in Scheme 1. First, BCT/CFO composite nanofibers are prepared by electrospinning based on the precursors prepared by the sol–gel method. Subsequently the fibers are dried under a magnetic field to form a film, which is then collected and pressed into a wafer. The wafer is sintered at 900 °C to promote the crystallinity of samples. The morphology, crystal structure, and thermal properties of the aligned BCT/CFO nanofibers are characterized by scanning electron microscopy (SEM), X-ray diffraction (XRD), and TG–DTA analysis. The effects of materials' compositions and microstructure on their magnetic properties, ferroelectric properties, and dielectric properties are studied. It is found that the dielectric constants of BCT/CFO composites with magnetic orientation are enhanced (by ∼1.5–3.5 times) as compared with BCT and BCT/CFO without magnetic orientation, although their remnant polarization are smaller than those of the non-orientated samples. The temperature-dependent dielectric loss (tan*δ*) for BCT/CFO composite is measured to reveal the interaction between CFO and BCT in the compound. The mechanisms of boosting the dielectric constants of BCT/CFO composites through a magnetic-field induction approach are elucidated, which have not been well revealed in previous work. This work not only provides a novel and effective approach in enhancing the dielectric constants of ceramic materials, which are much desirable for electronic applications such as multilayered ceramic capacitors, but also elucidates the interaction between ferromagnetic and ferroelectric phases in multiferroic compounds.

## 2 Experimental

### 2.1 Materials

All the chemical reagents used in the preparation of materials are of analytical grade. Barium acetate, calcium acetate, tetrabutyl titanate, ethylene glycol, and glacial acetic acid were purchased from Sinopharm Group Chemical Reagent Co., Ltd. CoFe_2_O_4_ material with an average nanoparticles diameter of ∼40 nm was purchased from Beijing Dk Nano Technology Co., LTD. PVP was purchased from Zhangjiakou Kerma Fine Chemicals Co., Ltd.

### 2.2 Preparation of BCT/CFO precursor sol

A certain amount of barium acetate were weighted, subsequently dissolved in hot aqueous solution of glacial acetic acid (36 wt% and 60 °C), and stirred for 30 min. Then, the corresponding amount of calcium acetate were added into the mixture and continuously stirred for 30 min. The resulting clear solution was cooled to room temperature. In the next step, viscous tetrabutyl titanate was added dropwise into the ethylene glycol methyl ether solution and stirred with a magnetic stirrer to obtain a clear solution, which was further slowly added dropwise to the above-mentioned acetic acid solution under magnetic stirring to obtain a pale yellow and transparent BCT solution. CoFe_2_O_4_ nanoparticles (with Co:Ti molar ratios of 1:3, 1:9, and 1:15) were added to the BCT solution. The mixture was heated and stirred with a magnetic stirrer at 80 °C for 20 min, then cooled to 40 °C, and subsequently stirred for 10 h. Finally 1 wt% of PVP was added at room temperature, and dissolved uniformly in the aforementioned mixture to obtain BCT/CoFe_2_O_4_ precursor sols.

### 2.3 Preparation of BCT/CFO composite fibers

The BCT/CoFe_2_O_4_ (BCT/CFO) precursor sol was processed into composite fibers by electrospinning at a spinning voltage of 20 kV. The as-prepared primary fibers were calcined at 500 °C and then ground to obtain fiber powders. The BCT/CFO composite fiber powders were pressed into wafers (Φ10 mm × 2 mm) and sintered at 900 °C for 3 h to form ceramic sheets for testing, which were labelled as BCT/CFO-x, where x = 0.0625, 0.1, 0.25 were the contents of CFO in the ceramics.

### 2.4 Preparation of aligned BCT/CFO composite fiber

At room temperature, the fiber powders were first dispersed in the PVP solution. The solution was dried under a magnetic field to form a film, which was then collected and pressed into a wafer. The wafer was sintered at 900 °C for 3 h to obtain the BCT/CFO ceramic sheet for testing, which was labelled as BCT/CFO-x-orientated.

### 2.5 Characterization

The microstructure and particle size distribution of the samples were observed by scanning electron microscope (SEM, Hitachi S-4300). The phase structure and crystal structure of the samples were studied by the X-ray diffractometer (Bruker Company, D8), using the Cu-Kα radiation (λ = 0.15418 nm). Thermogravimetric analysis (TGA Q50, Perkin–Elmer) was carried out in air to determine annealing conditions for the sample. Measurements on magnetic properties were performed on a vibrating sample magnetometer (VSM, Riken Denshi, BHV-525). Polarization-electric field (P-E) hysteresis loops were characterized using a standard ferroelectric tester (Radiant Precision, Germany). Measurement of the dielectric properties was performed by an impedance analyzer (WK5000B，UK). Capacitance-temperature (C-T) characteristics of the samples were measured using an LCR meter (HP 4284A, Agilent, Palo Alto, CA).

## 3 Results and discussion

### 3.1 Characterization of BCT/CFO composite samples

**Fig. 1:**
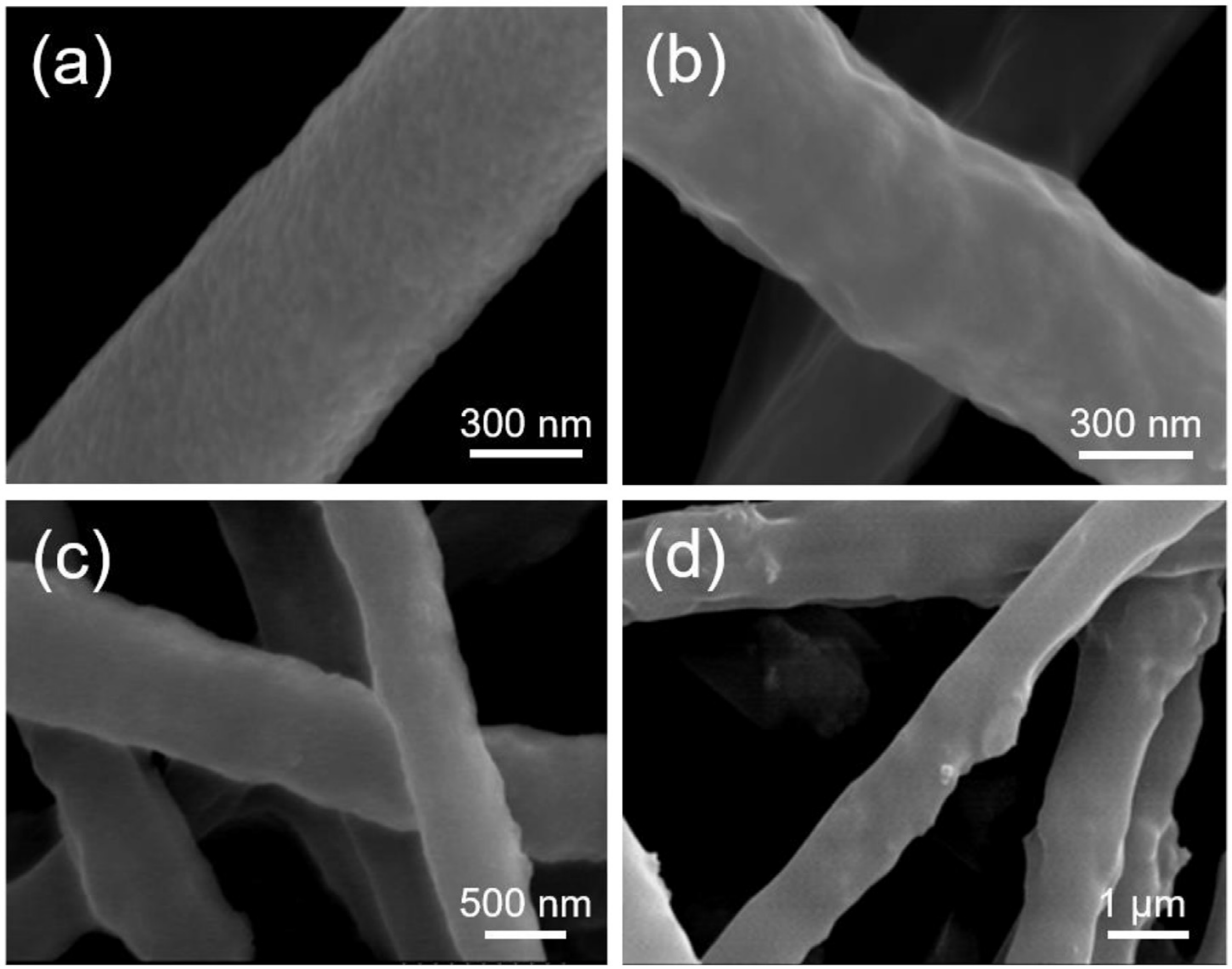
SEM images of the as-spun BCT/CFO-x composite fibers with different CoFe_2_O_4_ contents; (a) Pure BCT, (b) x = 0.0625, (c) x = 0.1, and (d) x = 0.25.

The CoFe_2_O_4_ nanoparticles used in this work are analyzed by SEM. The results are shown in Fig. S1. CoFe_2_O_4_ nanoparticles are mainly spherical and relatively uniform in morphology, with a primary diameter of ∼45 nm and an agglomerate diameter of ∼210 nm. The agglomeration occurs due to the intrinsic magnetic effects of CoFe_2_O_4_ nanoparticles. Fig. 1 shows the SEM images of the as-spun composite fibers prepared with different CoFe_2_O_4_ contents. As shown in Fig. 1a, the as-spun fiber of pure barium calcium titanate with a diameter of ∼625 nm has a smooth surface. Fig. 1b–d shows the as-spun composite fibers with different contents of cobalt ferrite, which have a diameter of ∼600–1500 nm. With increasing CFO content, the diameter of BCT/CFO as-spun fiber increases significantly. In the processing of precursors for electrospinning, magnetic stirring was kept to avoid the agglomeration of CoFe_2_O_4_ nanoparticles, which might result in uniformly distributed nanoparticles on the surfaces of BCT/CFO as-spun composite fibers, as shown in Fig. 1. In addition, it can be observed that the BCT/CFO as-spun fibers possess a dense structure before sintering.

**Fig. 2:**
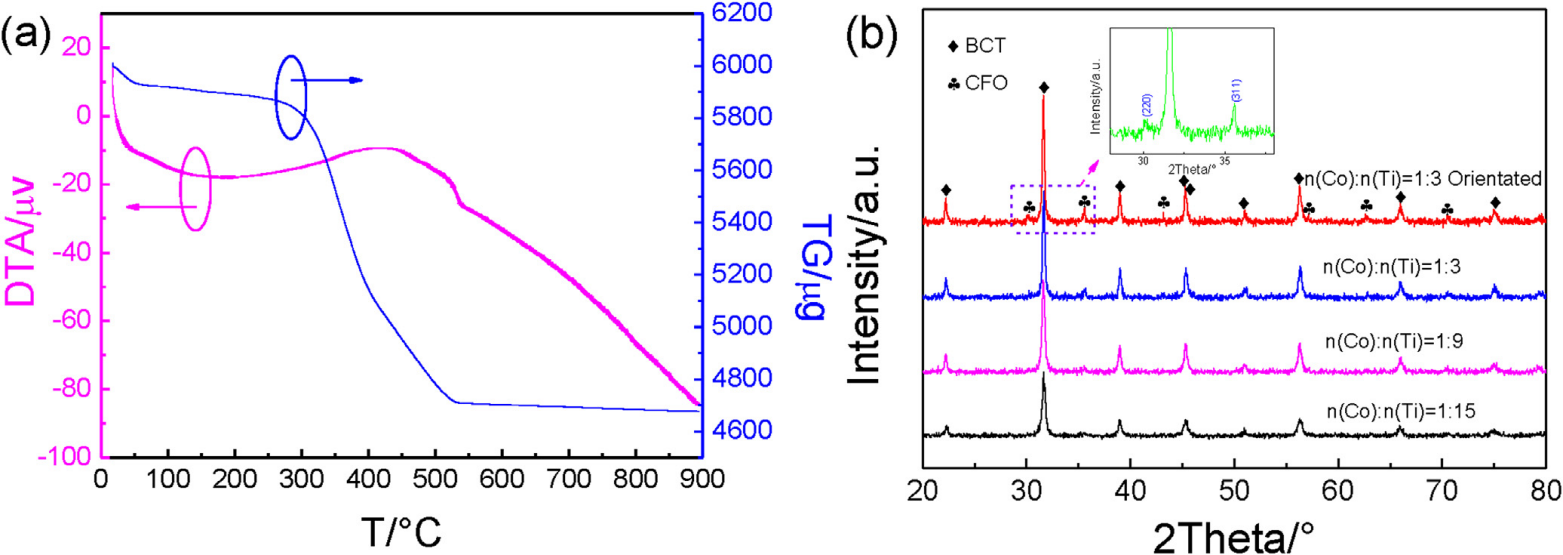
(a) Thermogravimetric analysis curve for BCT/CFO sol; (b) XRD patterns for fibers with different molar ratios of Co and Ti calcined at 900 °C.

Fig. 2a shows the thermogravimetric analysis curve of BCT/CFO as-spun fibers, indicating that the weight loss process of sample can be divided into three stages. The first stage is from room temperature to ∼276 °C with a mass loss of 2.5%, mainly due to the removal of physically absorbed water and a small amount of volatile organic matter. The second stage which is an exothermic reaction is in the temperature range of 276–528 °C with a mass loss of 19%, mainly due to the combustion of PVP. In the third stage, very small weight loss occurs in the temperature range of 528–900 °C. The TG curve becomes basically parallel to the temperature axis, indicating no further weight loss. The thermogravimetric analysis indicates that the heat treatment at 900 °C can completely remove the PVP ingredient of the as-spun fibers. The effects of sintering temperatures on XRD patterns of BCT/CFO composite fibers are shown in Fig. S2a. The diffraction patterns for samples sintered at 700 °C show that the main peaks correspond to whiterite-BaCO_3_ (CaCO_3_) and even CoTiO_3_ phases (PDF-# 15-0866), as marked in the XRD patterns. Other phases (e.g. Ba_2_TiO_4_), if any, are below the XRD detection limit. The results imply that BaCO_3_, CaCO_3_, and CoTiO_3_ may be the impurities. When the sintering temperature is increased to 800 °C, the strong diffraction peaks of (100), (220), (110), (311), (111), (400), (002), (200), (102), (211), (511), (440), (202), (533) and (103) crystallographic planes are observed, in consistent with those for BCT and CFO (PDF No: 80-1533 and 82-2234), respectively [31], [32]. After calcined at 900 °C, the XRD diffraction peaks become sharp, as shown in Fig. S2a. The XRD patterns for BCT/CFO composite fibers with different contents of cobalt ferrite calcined at 900 °C correspond well to CFO and BCT crystal phases. The XRD diffraction peaks can be indexed as the orthorhombic perovskite structure with a space group *Amm*2 for BCT (Fig. S2b) which is consistent with those reported by Acosta et al., [33] and the crystal planes of (220) and (311) for CFO (the inset in Fig. 2b), respectively. Fig. 2b also indicates that the BCT/CFO composites with less BCT contents have sharper diffraction peaks.

**Fig. 3:**
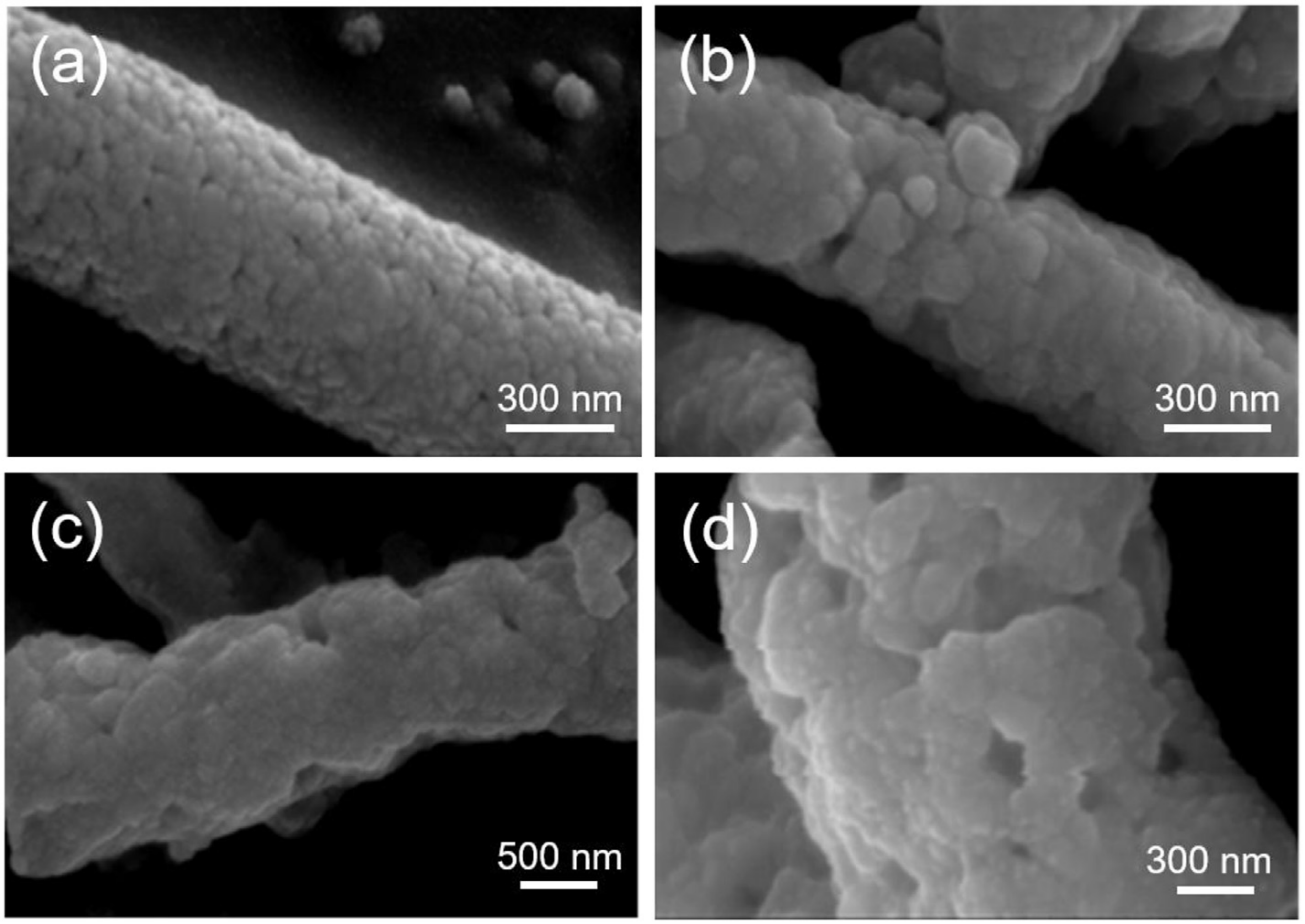
SEM images of BCT/CFO-x fibers calcined at 900 °C (a) Pure BCT, (b) x = 0.0625, (c) x = 0.1, and (d) x = 0.25.

Fig. 3 shows the SEM images of BCT/CFO composite fibers with different CoFe_2_O_4_ contents calcined at 900 °C. The fibers with different CFO contents are all found to have rough structures. It could be caused by the removal of PVP in the as-spun fibers when they were sintered at 900 °C. As compared to those of non-sintered samples shown in Fig. 1, the diameter of fibers does not significantly change after sintering. In addition, it is noted that the surfaces of sintered fibers with higher BCT contents are smoother and denser.

**Fig. 4:**
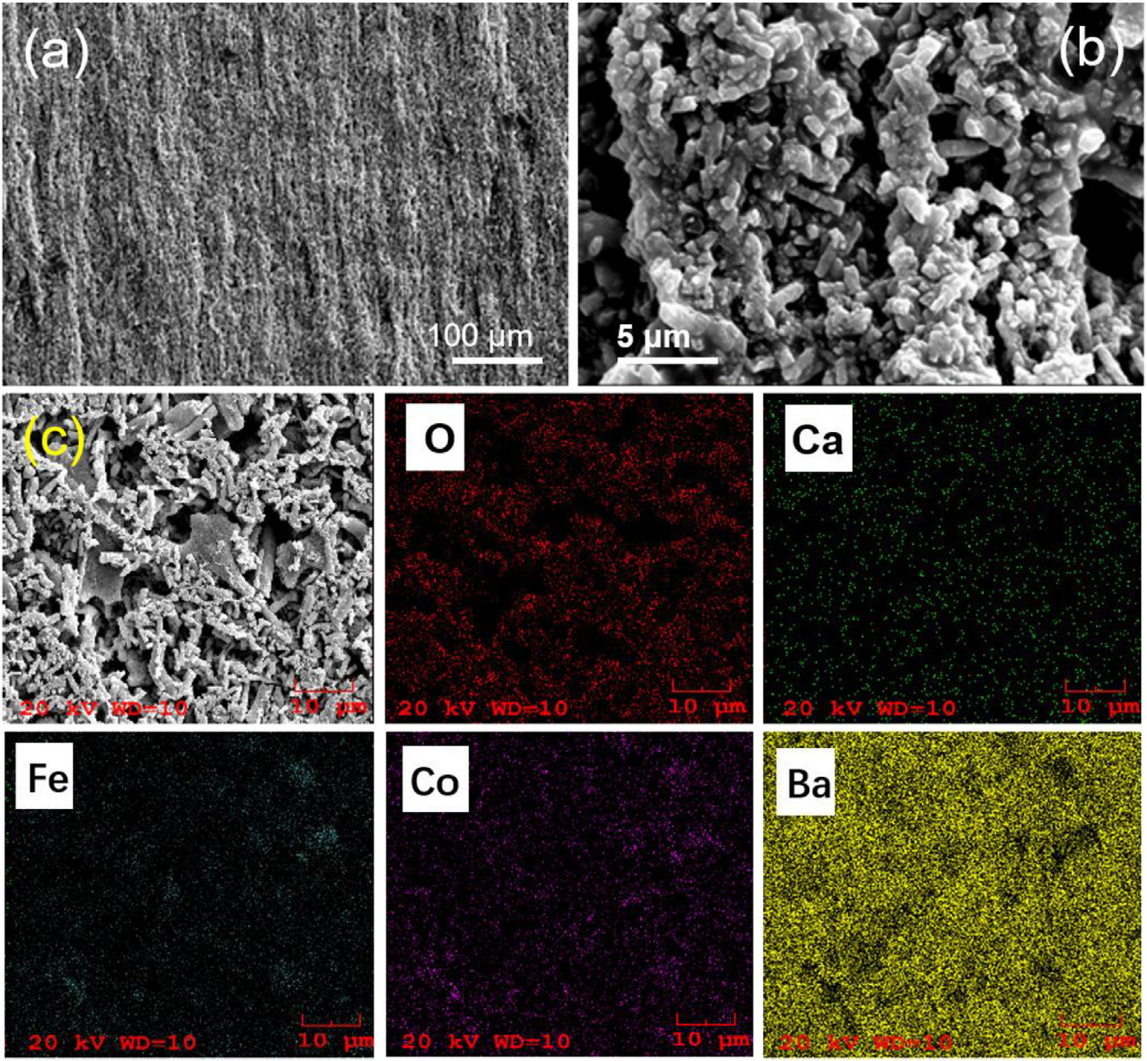
SEM images of films formed by drying BCT/CFO-0.25 under an external magnetic field. (a) low magnification; (b) high magnification. (c) SEM image of film sample and its corresponding EDS mapping patterns for O, Ca, Fe, Co, and Ba elements.

As illustrated in Scheme 1 the fibers with different CoFe_2_O_4_ contents were milled, pressed into pellets, and then sintered at 900 °C into ceramic wafers. Fig. S3 shows the SEM images of ceramic wafers. It is observed that the particle sizes of the BCT/CFO sample are slightly larger than that of the pristine BCT sample. It could be caused by the addition of CFO in the BCT matrix which reduces the melting temperature of BCT, facilitating the growth of BCT particles in the fibers. It is also observed that the fibers with higher content of BCT exhibit more continuous microstructures and could have higher density. The results indicate that BCT may facilitate the homogeneous distribution of liquid phase of fiber during the sintering processes. Specifically, the BCT/CFO-x fiber powders with x = 0.25 calcined at 900 °C were added to 10 wt% PVP and ethanol solution. The mixture was orientated under an external magnetic field and dried into a film. The morphology of obtained film was characterized by SEM, as shown in Fig. 4. The image clearly indicates one-dimensional orientation of fibers in the film and the fibers are aligned into bundles with a diameter of 4–6 μm. The orientation and alignment process could be related with the effects of applied magnetic field during drying. When the external magnetic field is applied, the BCT/CFO nanoparticles immersed in the liquid mixture could align with the magnetic field because of the interaction between the magnetic moments of CFO and the applied filed. Those BCT/CFO nanoparticles will eventually accumulate along the direction of the magnetic field. The uniform distribution of CFO and BCT particles inside the fibers is confirmed by energy dispersive spectrometer (EDS) mapping, as shown in Fig. 4c. The elements (oxygen, calcium, iron, cobalt, and barium) are found to distribute uniformly throughout the sample. This can be attributed to the advantages of the processing method.

### 3.2 Magnetic properties of BCT/CFO composite fiber

**Fig. 5:**
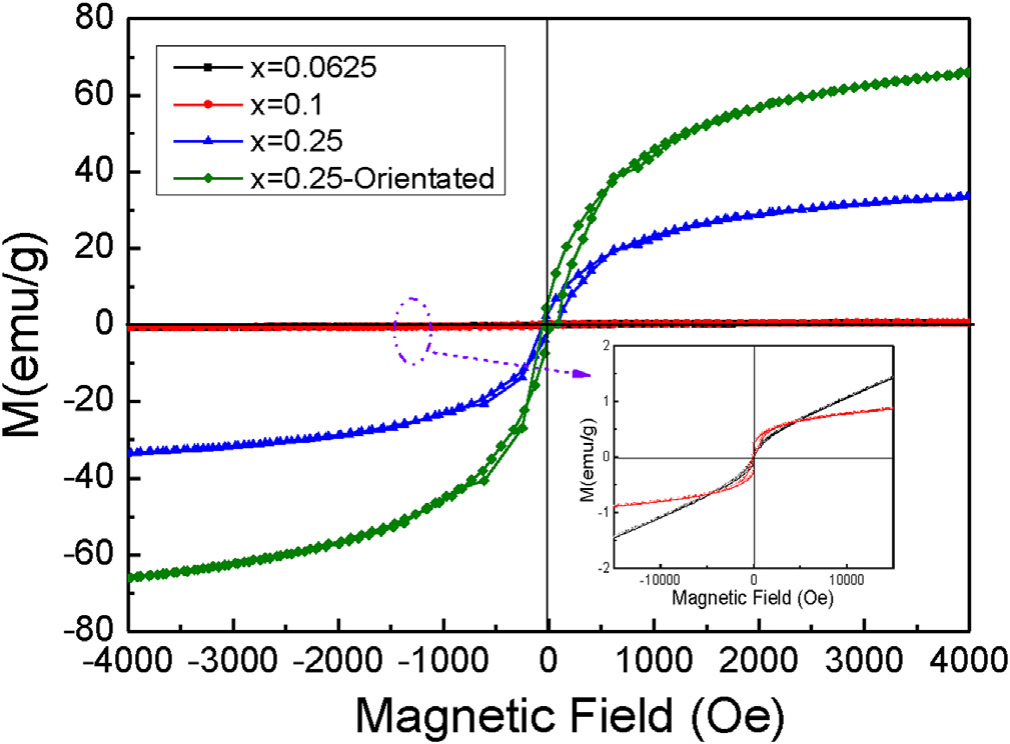
Magnetic hysteresis loops for BCT/CFO-x composite fibers with different contents of CoFe_2_O_4_. The inset is the magnification of plots for samples with x = 0.0625 and 0.1 (molar ratios Co: Ti = 1:15, and 1:9 respectively), The orientated composite fibers are measured under magnetic fields vertical to the orientation of fibers in the samples.

Fig. 5 shows the hysteresis loops for BCT/CFO-x composite fibers with different contents of CoFe_2_O_4_. For simplicity, the molar ratio Co:Ti of the sample is denoted as n(Co):n(Ti), and ‘*Orientated*’ represents that the composite fibers have been subjected to magnetic orientation treatment during processing. All the samples exhibit the characteristic hysteresis loops for typical soft magnetic materials, indicating that the magnetism of CFO is not affected by incorporating BCT in the fibers. The saturation magnetization of the composite fibers without magnetic orientation treatment much decreases with increasing BCT content (as shown in Fig. 5), which could mainly result from the fact that the continuity of ferromagnetic CFO phase or the magnetic ordering might be interrupted by the presence of BCT fine grains [32]. As a consequence, the regional magnetic moments significantly decrease with increasing BCT content. The saturation magnetization of the orientated composite fibers measured under a magnetic field vertical to the fiber orientation is found to be dramatically higher than that of composite fibers without magnetic orientation treatment. Magnetic anisotropy can be clearly observed in the composite fibers with magnetic orientation treatment.

### 3.3 Polarization–electric field hysteresis loops for BCT/CFO composite fibers

**Fig. 6:**
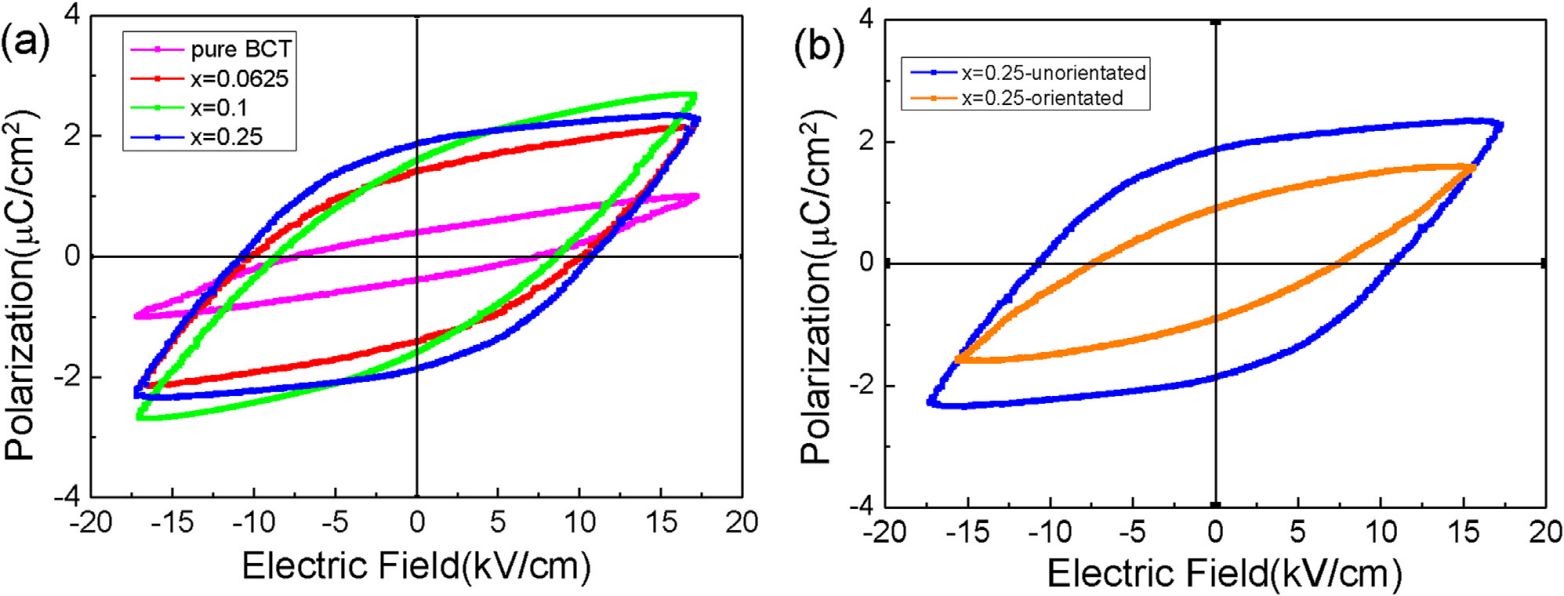
Ferroelectric hysteresis loops for BCT/CFO-x composite fibers (a) with different amounts of CoFe_2_O_4_ and (b) with and without magnetic orientation treatments. x = 0.0625, 0.1, and 0.25 correspond to molar ratios Co:Ti = 1:15, 1:9, and 1:3, respectively.

Fig. 6 shows the typical ferroelectric hysteresis (P-E) loops for BCT/CFO-x composite fibers with different amounts of CoFe_2_O_4_. In Fig. 6a, it is indicated that the composite fibers could exhibit ferroelectricity. The remnant polarization, saturation polarization and coercive field of the sample all increase with increasing BCT content. Fig. 6b shows the P-E loops of composite fibers with and without magnetic orientation treatments. It can be found that the coercive field, the saturation polarization and the remnant polarization of the sample with magnetic orientation treatment are all smaller than those of sample without magnetic orientation treatment.

### 3.4 Dielectric properties of BCT/CFO composite fibers

**Fig. 7:**
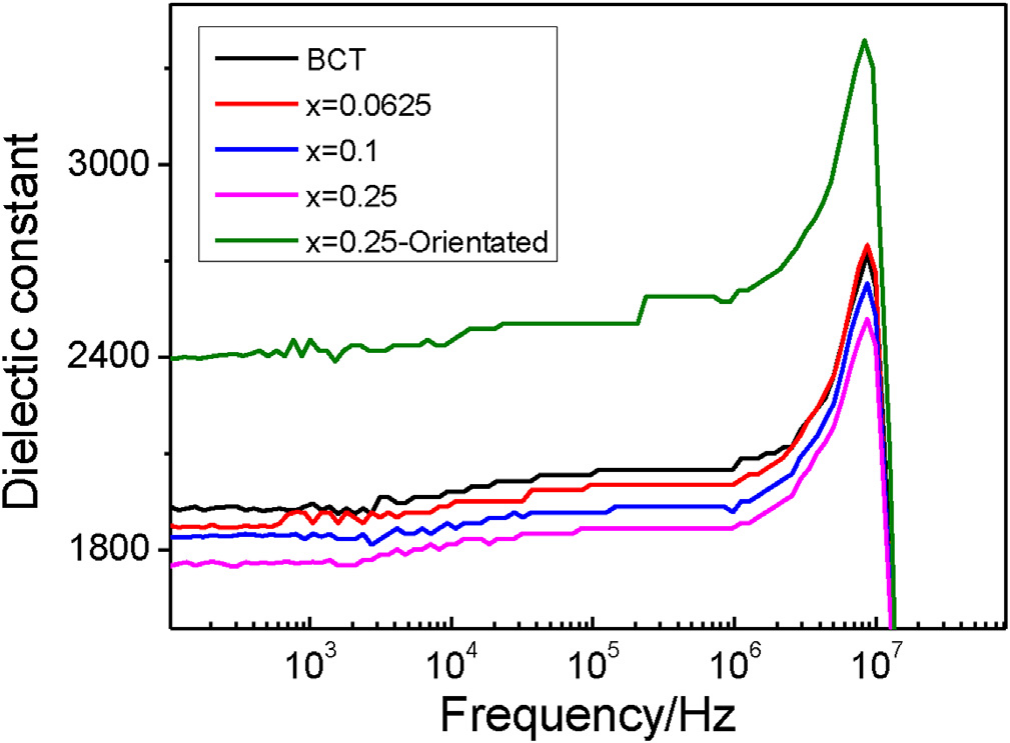
Dielectric spectrum for BCT/CFO composite fibers at room temperature; x = 0.0625, 0.1, and 0.25 correspond to molar ratios Co:Ti = 1:15, 1:9, and 1:3, respectively.

Fig. 7 shows the (real component) dielectric spectrum of the BCT/CFO-x composite fibers at room temperature, in the frequency range of 20 Hz–15 MHz. It is found that the dielectric constant of BCT/CFO-x composite fibers decreases obviously with increasing CFO content x. The XRD analysis has indicated that the BCT/CFO composite fibers are a two-phase system, in which CFO has a resistivity of ∼10^7^ Ωm, thus belonging to a semiconductor, whereas BCT has a resistivity of ∼10^12^ Ωm and is an insulator [34]. Therefore, BCT has a higher dielectric constant than CFO. Since BCT has a content of ∼75–93.7 wt% and acts as a continuous phase or a matrix, the addition of CFO can decrease the dielectric constant of the two-phase composite. Thus the dielectric constant decreases with increasing CFO content, which is consistent with those reported by Qu et al. [34]. It is also found that within the frequency range of 20 Hz–15 MHz, the dielectric constant of BCT/CFO composite fibers slightly increases with increasing frequency. When the frequency reaches 8.68 MHz, the dielectric constant of BCT/CFO composite fibers exhibits a resonance peak, and further decreases rapidly with increasing frequency. The dielectric constants of BCT/CFO-x composite fibers with different CFO contents exhibit similar natural resonant frequencies. For the ceramic dielectric materials, the non-uniformity in materials structure causes the non-uniformity in dielectric and electrical conductivity. Under the external electric field, charges accumulate at the grain boundaries of dielectric material and then result in Maxwell–Wagner interfacial effect [35]. When the testing frequency is close to the natural resonant frequency, the grain boundaries, dislocations, and surface lattice defects in dielectric materials have enough time to accumulate a large number of induced charges, resulting in higher dielectric constants. When the frequency of electric field is higher than the resonant frequency, however, the interfacial accumulation rate of charges does not meet the varying speed of electric field, resulting in lower dielectric constants. Most importantly, it is observed that the dielectric constants of BCT/CFO-0.25 composite fibers with magnetic orientation are significantly enhanced (by ∼1.5–2 times) as compared with those of BCT and BCT/CFO-x without magnetic orientation. For the BCT/CFO-0.0625 sample (that is n(Co): n(Ti) = 1:15), the oriented one has a dielectric constant ∼3.5 times as large as that without magnetic orientation, as shown in Fig. 7 and Fig. S4.

### 3.5 Temperature dependence of the dielectric loss (tan δ) of BCT/CFO composite fibers

**Fig. 8:**
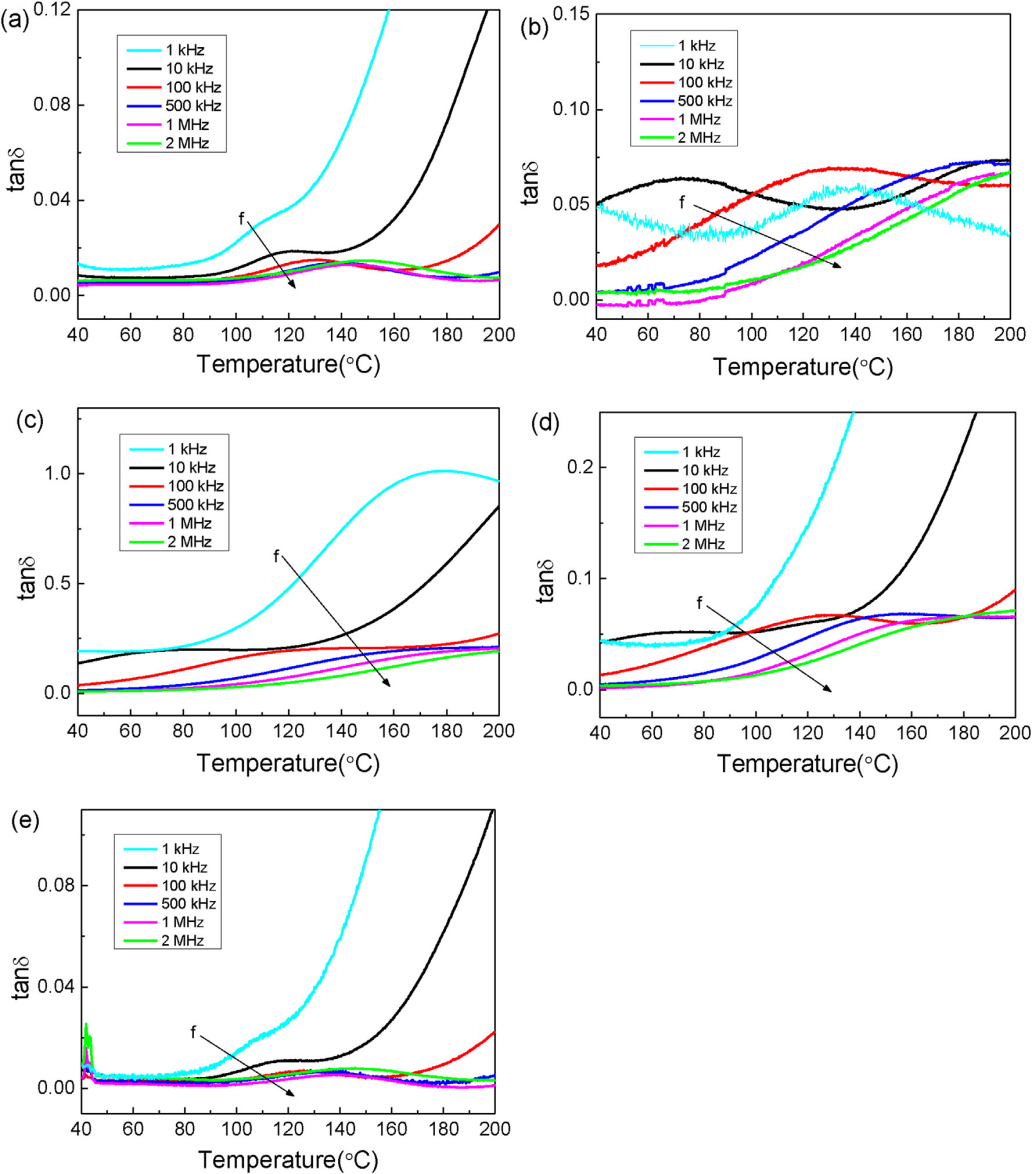
Temperature dependence of the dielectric loss (tan δ) for BCT/CFO-x composite fibers measured at different frequencies (a) pure BCT, (b) x = 0.25 (Co:Ti = 1:3), (c) x = 0.25 (Co:Ti = 1:3), orientated, (d) x = 0.1 (Co:Ti = 1:9), and (e) x = 0.0625 (Co:Ti = 1:15).

Fig. 8a–e shows the temperature-dependent dielectric loss (tan*δ*) for BCT and BCT/CFO-x composite, respectively, which is measured at different frequencies. For BCT, tan*δ* measured at 10 kHz exhibits a maximum at around 121.2 °C, which corresponds to the ferroelectric-to-paraelectric phase transition temperature or Curie temperature T_c_. The BCT/CFO-x samples show different dielectric behaviors with different CFO contents *x*. The peak value of dielectric loss and T_c_ are listed in Table 1. The Curie temperature T_c_ is found to decrease significantly with increasing CFO content. Meanwhile, the dielectric losses at T_c_ for BCT/CFO-x samples are larger than that for BCT. For the BCT/CFO-0.25 composites, the sample with magnetic orientation has obviously higher T_c_ and tan*δ* as compared with that without magnetic orientation.

**Table 1:** Dielectric and ferroelectric properties of BCT/CFO-x composite ceramics.

	x = 0	x = 0.0625 un-orientated	x = 0.10 un-orientated	x = 0.25 un-orientated	x = 0.25 orientated
ε_r_	1954	1903	1865	1712	2415
tanδ at 10 kHz	0.0188	0.0642	0.0523	0.0640	0.2010
E_a_ (eV)	2.67	2.75	0.61	0.47	0.48
T_c_ (°C) at 10 kHz	121.2	118.0	75.9	73.3	81.5
P_r_ (μC/cm^2^)	0.38	1.37	1.57	1.86	0.86

**Fig. 9:**
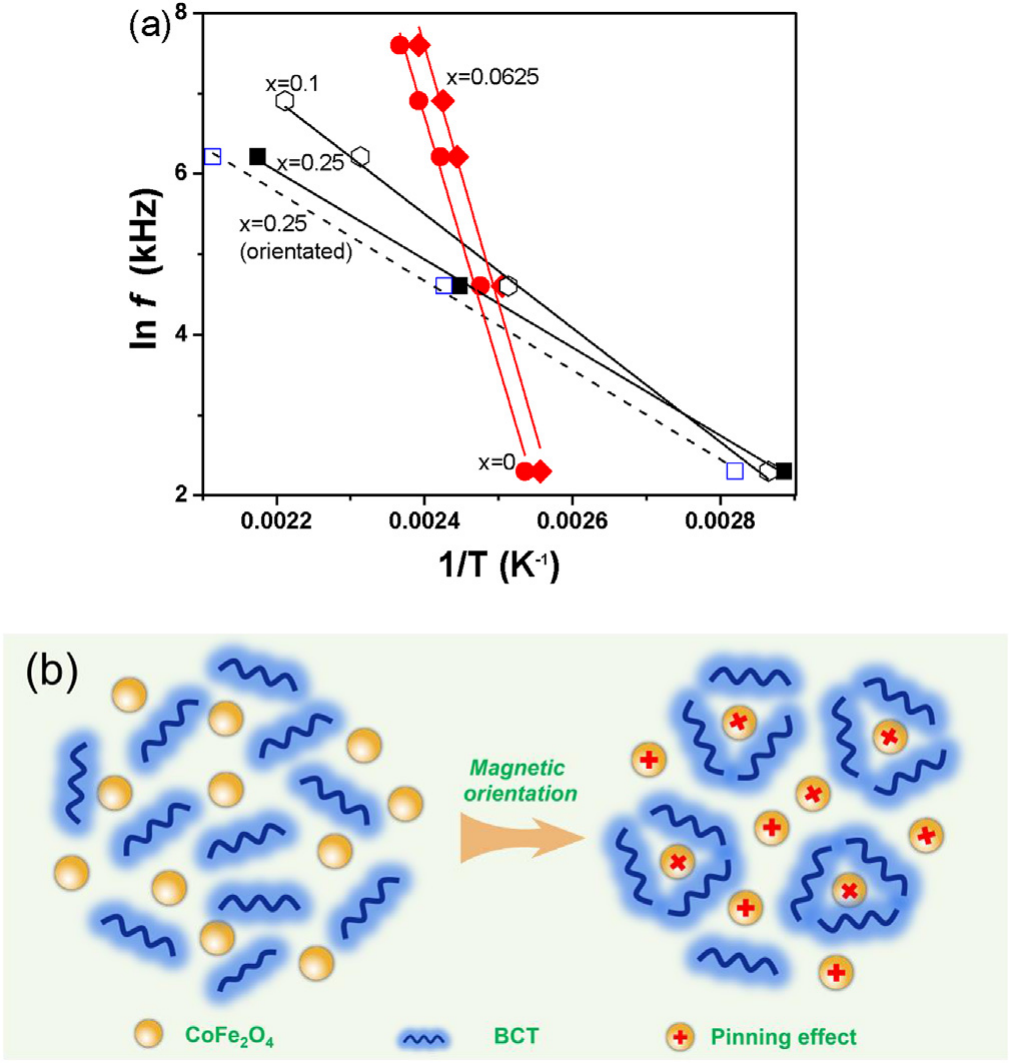
(a) Arrhenius plots for the dielectric relaxation in BCT/CFO-x composite ceramics with different CFO contents x. (b) Illustration of ordering of polar domains in BCT before and after the magnetic orientation of CFO nanoparticles, corresponding to the weak and strong pinning effects of CFO on the polar domains, respectively.

The dielectric relaxation of BCT ceramics and BCT/CFO-x composite ceramics can be well described by the Arrhenius relation as follows [36],

(1) $$f=f_{0}\cdot e^{-E_{a}/k_{B}T}\text{,}$$

where f_0_ is an attempt frequency, E_a_ is the apparent activation energy characterized the relaxation process. The Arrhenius plots for BCT and BCT/CFO-x samples are shown in Fig. 9a. The fitting of activation energy by Eq. (1) is listed in Table 1. The activation energy for BCT ceramics or BCT/CFO-x composite ceramics with low CFO contents (x = 0.0625) is about 2.6–2.7 eV, which is typical for the relaxation of domain walls in ferroelectric phases close to T_c_. However, the activation energy for BCT/CFO-x composite ceramics with high CFO contents (x > 0.0625) is much lower than 1.0 eV, suggesting that the nature of dielectric relaxation could be related with ions such as oxygen ions or atomic defects.

In particular, the dielectric loss of BCT/CFO-0.25 with magnetic orientation is much higher than that of BCT/CFO-0.25 without magnetic orientation, suggesting that the applied magnetic field could lead to the ordering of those oxygen ions or atomic defects. Judging the fact that T_c_ for BCT/CFO-0.25 with magnetic orientation is much higher than that without magnetic orientation, it is thus envisaged that the ordered oxygen ions or atomic defects could act as pinning centers for domain-wall motions or rotations.

### 3.6 Mechanisms of enhanced dielectric properties of BCT/CFO composite fibers

As shown in Fig. 7, the BCT/CFO composite ceramics without magnetic orientation mainly show the dielectric constant-frequency characteristic of BCT, while the influence of CFO ingredient on the relationship between dielectric constant and frequency is relatively weak. Considering the fact that the dielectric constant of BCT/CFO is mainly attributed to the ferroelectric BCT phase, the effect of CFO content on the dielectric constants of those BCT/CFO composites could be also weak at the frequency ranging from 20 Hz to 15 MHz. For the magnetic-field orientated BCT/CFO composite ceramics with 25 wt% CFO, although the dielectric constant-frequency characteristic is similar to that for the BCT/CFO composite ceramics without magnetic orientation, its dielectric constants are significantly boosted at all testing frequencies as compared with those of BCT/CFO composite ceramics without magnetic orientation. Therefore, the effect of orientated magnetic CFO phase on the dielectric constants of ferroelectric BCT phase could be significant.

In macroscopic points of view, the dielectric constant ε_r_ of an isotropic dielectric material is defined by the following equation [37].

(2) $$P={\text{ε}}_{0}\text{χ}E\text{,}$$

where **P** is the macroscopic polarization, **E** is the applied electric field, χ is the susceptibility, ε_0_ is the vacuum dielectric constant and ε_r_ = 1+χ. Since the crystalline solids have different dielectric characteristics along different crystallographic orientations, the dielectric constant depends on the arrangement of molecules or ions in the crystals. In general, the direction of polarization and the applied electric field are not the same, therefore, ε_r_ is determined by the magnitude of **P** which is an average over all polarization orientations in a dielectric material. In the BCT/CFO composite fibers without magnetic orientation the fibers are randomly distributed, so as the polarizations of BCT/CFO fibers. Under a magnetic field applied along the sample surface (in the lateral direction), the BCT/CFO fibers are aligned along the lateral direction, as shown in Fig. 4. By applying an external electric field **E** perpendicular to the sample surface, the magnitude of **P** for the aligned BCT/CFO fibers should be larger than that for the un-aligned fibers, resulting in the obviously enhanced ε_r_.

The enhanced dielectric constant of BCT/CFO composite ceramics with magnetic orientation could be also explained by a microscopic mechanism based on the magnetic, ferroelectric and dielectric analyses described in Sec.3.2–3.5. Due to the electro-magneto-coupling, the CFO nanoparticles in the BCT matrix could act as pinning centers for the polar domains in BCT, as illustrated in Fig. 9b. Basically, the motions or rotations of domain walls of polar domains in the ferroelectric BCT regions could be hindered by the magnetic CFO nanoparticles due to the electro-magneto-coupling effect. In the BCT/CFO composite ceramics without magnetic orientation, it is suggested that the un-orientated CFO ferrite nanoparticles could spatially interrupt the ordering of ferroelectric BCT phase although the electro-magneto-coupling between CFO and BCT might be weak [38], as illustrated in Fig. 9b. In the BCT/CFO composite ceramics with magnetic orientation, the magnetic ordering of CFO phase driven by an applied magnetic field could lead to the enhanced local magnetic fields around the CFO nanoparticle. As a result, the electro-magneto-coupling between CFO nanoparticles and the polar domains in BCT is increased. Consequently, the ordering of ferroelectric phase of BCT could be weakened [39]. Such effect of CFO on the ferroelectric properties of BCT is well demonstrated by the ferroelectric P-E loop results (Table 1), as shown in Fig. 6, which indicate the significantly reduced saturation polarization and remnant polarization of the BCT/CFO-0.25 composite ceramics with magnetic orientation as compared with those of ceramics without magnetic orientation. On the other hand, such pinning effects of CFO nanoparticles on the ferroelectric properties of BCT are also consistent with the results of dielectric relaxation, which indicates the significantly increased dielectric loss of ferroelectric-to-paraelectric transition in BCT/CFO-0.25 composite ceramics with magnetic orientation as compared with those of ceramics without magnetic orientation. Therefore, the strong pinning effect of magnetic-orientated CFO nanoparticles on the motions or rotations of domain walls of polar domains in the ferroelectric BCT regions leads to the increased dielectric susceptibilities of BCT/CFO composite ceramics with magnetic orientations, which well explains their enhanced dielectric constants.

## 4 Conclusions

In summary, BCT/CFO composite fibers with enhanced dielectric constants were prepared by a combination of electrospinning and magnetic orientation approach. The effects of magnetic field orientation and CFO contents on the magnetic, ferroelectric and dielectric properties were studied. The BCT/CFO composite ceramic exhibits huge magnetic anisotropy. The dielectric constant of BCT/CFO composite ceramic with magnetic orientation is 1.5–3.5 times as high as that without magnetic orientation. This study thus demonstrates that the magnetic-field induced orientation in multiferroic composites could be facile and effective in boosting their dielectric constants, providing a novel technology to further enhance the dielectric properties of lead-free ferroelectric materials.

## Appendix A Supplementary data

mmc1 mmc1
