# Supplementary material for: Magnetic-field-induced dielectric behaviors and magneto-electrical coupling of multiferroic compounds containing cobalt ferrite/barium calcium titanate composite fibers
Source: J Alloys Compd. 2018 Apr 5;740:1067–76. doi: 10.1016/j.jallcom.2018.01.081 (PMC5806601; doi:10.1016/j.jallcom.2018.01.081)
Supplement: mmc1 [file mmc1.doc]

Supporting Information

**Magnetic-field-induced dielectric behaviors and magneto-electrical coupling of** **multiferroic compounds containing cobalt ferrite/barium calcium titanate composite fibers**

Deqing Zhang ^a,#^, Junye Cheng ^b,c,#^, Jixing Chai ^a^, Jiji Deng ^a^, Ran Ren ^c^, Yang Su ^b^, Hao Wang ^b^,*, Chunqing Ma ^c^, Chun-Sing Lee ^c^, Wenjun Zhang ^c^, Guangping Zheng ^d^,*, Maosheng Cao ^e^

^a^ *School of Materials Science and Engineering, Qiqihar University, Qiqihar 161006, China*

^b^ *Guangdong Provincial Key Laboratory of Micro/Nano Optomechatronics Engineering, College of Mechatronics and Control Engineering, Shenzhen University, Shenzhen 518060, China*

^c^ *Center of Super-Diamond and Advanced Films (COSDAF), City University of Hong Kong, Hong Kong 999077, China*

^d^ *Department of Mechanical Engineering, Hong Kong Polytechnic University, Hung Hom, Kowloon, Hong Kong*

^e^ *School of Materials Science and Engineering, Beijing Institute of Technology, Beijing 100081, China*

# Junye Cheng and Deqing Zhang contributed equally to this work

*Corresponding author.

*E-mail addresses*: mmzheng@polyu.edu.hk (G.P. Zheng), whao@szu.edu.cn (H. Wang).


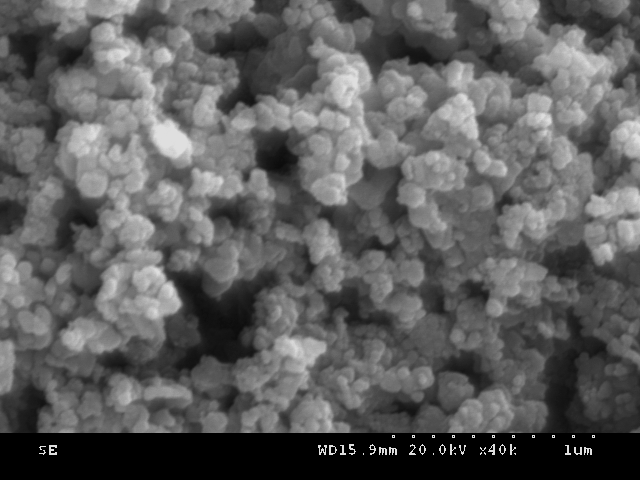


**Figure S1**. SEM image of CoFe_2_O_4_ nanoparticles used for the preparation of CFO/BCT precursor sol.


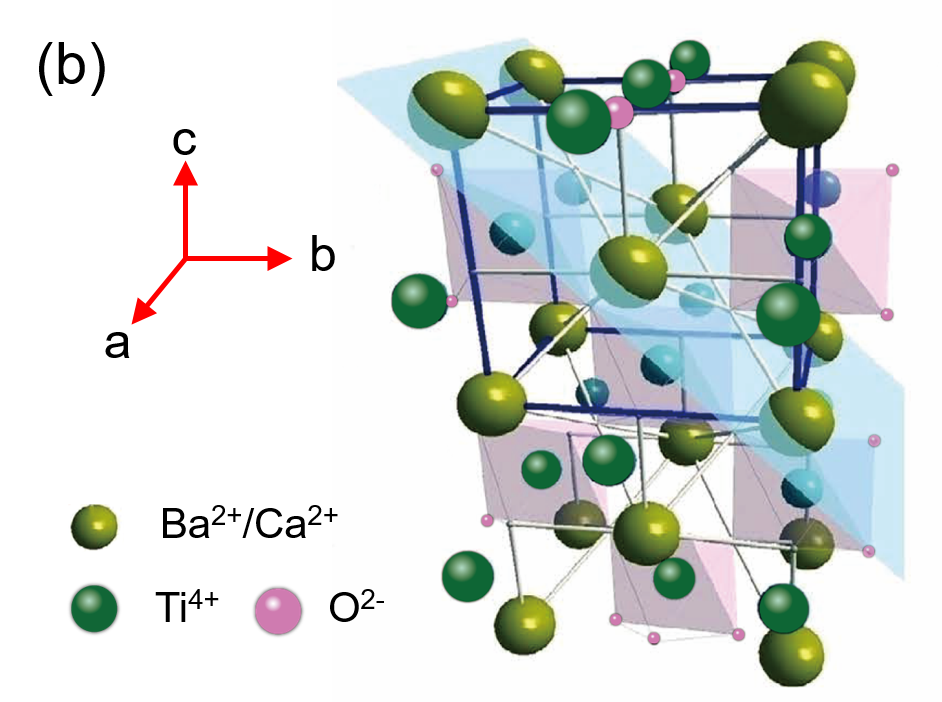


**Figure S2.** XRD diffraction patterns of fibers (n(Co):n(Ti)= 1:3) calcined at different temperatures.


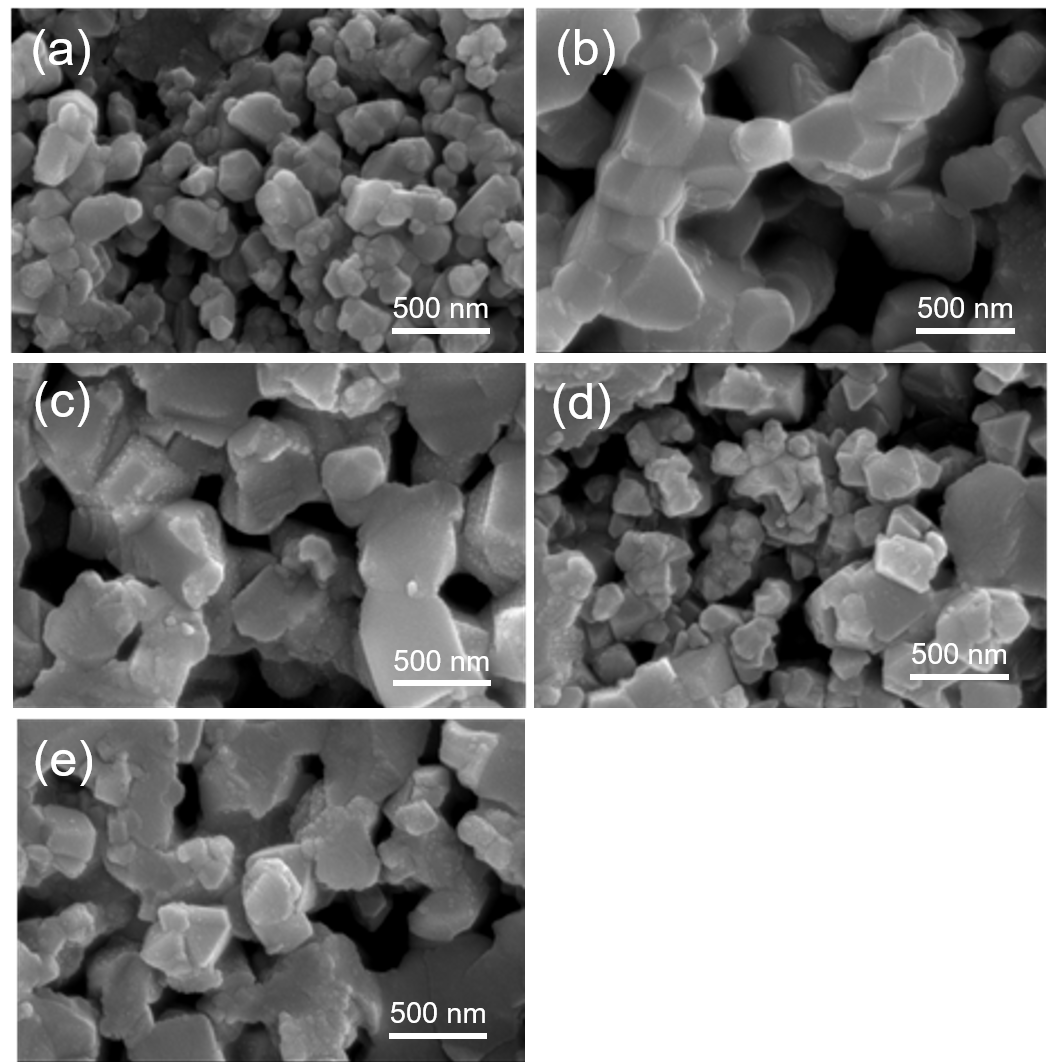


**Figure S3**. SEM images of BCT/CFO-x ceramic wafers with different CoFe_2_O_4_ contents calcined at 900 °C (a) Pure BCT, (b) x=0.0625, (c) x=0.1, and (d) x=0.25, and (e) x=0.25, orientated.

**Figure S4.** Dielectric spectrum of CFO/BCT composite fibers at room temperature.
